# Can taking the perspective of an expert debias human decisions? The case of risky and delayed gains

**DOI:** 10.3389/fpsyg.2014.00989

**Published:** 2014-09-04

**Authors:** Michał Białek, Przemysław Sawicki

**Affiliations:** Department of Economic Psychology, Centre for Economic Psychology and Decision Sciences, Kozminski UniversityWarsaw, Poland

**Keywords:** risk, intertemporal choices, expert, dual-process theory, decision-making

## Abstract

In several previously reported studies, participants increased their normative correctness after being instructed to think hypothetically, specifically taking the perspective of an expert or researcher (Beatty and Thompson, 2012; Morsanyi and Handley, 2012). The goal of this paper was to investigate how this manipulation affects risky or delayed payoffs. In two studies, participants (*n* = 193) were tested online (in exchange for money) using the adjusting procedure. Individuals produced certain/immediate equivalents for risky/delayed gains. Participants in the control group were solving the problem from their own perspective, while participants in the experimental group were asked to imagine “what would a reliable and honest advisor advise them to do.” Study 1 showed that when taking the perspective of an expert, participants in experimental group became more risk aversive compared to participants in the control group. Additionally, their certain equivalents diverged from the expected value to a greater extent. The results obtained from the experimental group in Study 2 suggest that participants became less impulsive, which means they tried to inhibit their preferences. This favors the explanation, which suggests that the perspective shift forced individuals to override their intuitions with the social norms. Individuals expect to be blamed for impatience or risk taking thus expected an expert to advise them to be more patient and risk aversive.

## INTRODUCTION

Human life presents continuous choices. In this text, we will focus on choices made in risk conditions (sticking to a permanent post vs. starting your own business) and intertemporal ones (buying an iPad vs. saving money). Studies have shown that people make mistakes in both areas, which can lead to serious social problems, such as gambling or obesity.

Overwhelming media advertisements and marketing strengthen impulsive behaviors in the modern society. This results for an example in obesity and financial debts. Nowadays, people are often facing artificial risky problems (stock market, inflation) for which they are not prepared (for extended review, see Todd and Gigerenzer, 2012). This suggests a need to provide support to people so they can make more rational decisions, especially those that are intertemporal and risky.

One of the recently introduced methods to support thinking, specifically reasoning, is hypothetical thinking. People are asked to assess a problem from the perspective of an expert or researcher (Beatty and Thompson, 2012; Morsanyi and Handley, 2012). This usually results in increased normative correctness of their mental processing. Our aim is to test this method in a new field of cognition, that is, decision-making under risk and delay. We hope to validate the method of taking the perspective of an expert as an efficient debiasing method.

### DECISIONS UNDER RISK

Uncertainty about the future is an inherent part of human existence. While there are events we can be sure of and others that are impossible to predict, most of us have to deal with probabilistic situations. Studies on choices in risky conditions show that people have difficulty understanding information about probabilities. In a classic study, Tversky and Kahneman (1971) showed that when people assess the probability of events, they tend to ignore base rate information and instead rely on the social stereotype.

One of the main assumptions of prospect theory is that in risky situations, people underestimate moderate and large probabilities but overrate rare events (Kahneman and Tversky, 1979). Failure to understand the rules of probability theory as well as the fact that people overrate small probabilities are possible causes for the high percentage of people participating in different types of gambling. Despite the unfavorable profit-to-risk ratio, studies have shown that 82% of adult Americans (Welte et al., 2002), 72% of Canadians (Azmier and Clements, 2001), and 68% of adult British citizens (Wardle, 2007) admit to gambling. Even part of the stock market investors treat investing as a substitute of gambling. (Markiewicz and Weber, 2013).

Biased perception of randomness is a challenge in the healthcare domain. For example, in the medical context, there is the question of informing patients about the probability of various diseases. Much empirical evidence has shown that people have serious problems estimating small probabilities. In particular, people are insensitive to changes in the magnitude of these probabilities (Kunreuther et al., 2001; Siegrist et al., 2008; Tyszka and Sawicki, 2011).

### INTERTEMPORAL CHOICES

Intertemporal choices present people with different challenges. In everyday life and in politics or economic affairs, some decisions are based on choosing between payments that occur at a time different from the time when the decision is made. For example, deciding whether to eat fast food immediately or wait for a balanced meal is based on the same psychological mechanisms as deciding whether to spend your profit immediately or invest it. The issue of intertemporal choices is also examined in terms of self-control, for example, not succumbing to temptation. In the classic research commonly known as the marshmallow test, Walter Mischel offered a four-year-old child a sweet and said if the child decided not to eat it, he or she would soon get two marshmallows. The experimenter then left the room, leaving the child alone with the temptation. If the child did not wait, he or she got only one marshmallow instead of two. The results showed that people who had greater self-control when they were children, scored higher on the SAT test several years later, exhibited fewer behavioral problems, coped better with stress, and were more focused and attentive (Mischel et al., 1989).

The inability to defer gratification may also lead to serious social problems, such as obesity. Obesity is estimated to be the seventh leading cause of mortality in the world (Ezzati et al., 2002). In 2007–2008, 68% of adults in the US were overweight, and 33.8% of them were obese (Flegal et al., 2010).

### SOCIAL NORMS AND NORMATIVE MODELS

For many cognitive processes there is a normative model, which states what is correct (e.g., logic for reasoning). For risk taking, multiplication of gain and probability of its occurrence expected value (EV) is used as a normative model. Those who expect more for a lottery than its EV are overly risk seeking, and those who expect less thank the EV are risk averse. The intertemporal choices do not have a normative model, but because of changes in our societies and extended lifespan, patience (to some degree) is seen as rational.

Social norms also regulate the behavior. Typically, patience and the ability to avoid acting impulsively are virtues (Haidt and Joseph, 2004). Children are rewarded when they show the ability to postpone reward.

Rational, according to the normative model of risk taking, would be to take a well calculated risk. It is unknown whether there are any consistent social norms regarding risk-taking, but experiencing a loss because of risk-taking (action) is blamed more than missing a chance to profit (omission, Ritov and Baron, 1990, 1995). This happens because people expect to be blamed when taking the risk, and risk avoidance can be seen as a socially accepted behavior.

### METHODS IMPROVING DECISION MAKING

Some studies have focused on debiasing individuals in their conclusions and decisions. These studies introduced different types of instruction or additional information to help people override their initial, biased intuitions. There are two, usually implicit, assumptions behind these manipulations.

First group of researchers tries to inform people about the normative models and procedures (presenting people with the concept of validity, EV or base rates). They assume that people are making mistakes because they lack the appropriate knowledge or intuitions regarding the field of probability or logic (or mindware, as called by Stanovich, 2009b). In this view, an efficient method of debiasing would be a request to rely on a specific, formal procedure, e.g., “being presented with the concept of logical validity, please try to assess following conclusions according to their validity” (Evans et al., 1993).

The other group of researchers believes that biased thinking is not a result of the lack of appropriate knowledge but of cognitive miserliness (Fiske and Taylor, 1991), which means that individuals are making biased decision because of lack of available cognitive resources and/or motivation to use reflexive processing. When motivated and having enough time, individuals show less biased decision-making. In this view, an efficient method of debiasing is an instruction that relates to the procedure, supporting a deeper and reflexive thinking. An example of such instruction would be, “please try to override your initial beliefs and focus on the logical structure of presented problems,” like used by Moutier et al. (2002).

Both approaches did not produce any satisfying and consistent increase in normative correctness of decisions. Despite the consensus in the literature that debiasing requires decoupling of the intuitions with effortful processing (Croskerry et al., 2013), it seems that people are having troubles willingly override their initial beliefs, even when instructed to do so and when they are motivated and have appropriate knowledge.

In contemporary literature, we can also find other methods of improving individual’s cognitive processing. Hypothetical thinking can increase the normative correctness of decisions by increasing chances of using effortful, rule based processing (called Type 2, Evans and Over, 1996; Evans, 2007) but also inhibiting intuitive and heuristic responses (Type 1 processing). For example, Loewenstein et al. (2001) instructed participants to imagine the consequences of both presented alternatives, and thus debiased people from the vividness effect. Lord et al. (1984) instructed their participants to imagine the opposite when considering social dilemmas (e.g., capital punishment). Thanks to this strategy, they improved the objectivity of their judgments. Baron (2008) proposed open-minded thinking to help override simple heuristic-cued intuitions. Trippas (unpublished doctoral dissertation) showed that cognitive style, understood as willingness to engage in Type 2 processing rather than cognitive ability, influences accuracy of reasoning. This suggests that human cognition can be improved by encouraging people to think hypothetically (Type 2 processing).

Considering a problem from an expert’s perspective is quite a natural instruction. People typically perceive experts as unbiased and reliable sources of information. When taking an expert’s perspective, intuitions and emotions should play a minor role, and thanks to this manipulation, reflexive processing (1) should be used more often compared to standard cases and (2) should override the internal conflict with intuitions.

In some studies, the instructing participants to take the experts perspective effectively encouraged the use of Type 2 processing, leading to less biased human performance (see Greenhoot et al., 2004; Thompson et al., 2005; Ferreira et al., 2006; Beatty and Thompson, 2012). However, when testing children, the effect of the instruction was ambiguous. The instruction, “please answer the questions taking the perspective of a perfectly logical and rational person (pp. 328),” as used in the study of Chiesi et al. (2011), seemed to work only for high cognitive ability individuals. This is possibly a result of the lack of learned rules and procedures, which could be applied to the task in the reported study or lack of cognitive resources. We can assume that the rules of thinking, just like driving a car, require much more cognitive resources when they are just learned while requiring fewer resources with greater practice (Stanovich, 2009a).

### QUESTIONS AND HYPOTHESIS

As stated in the introduction section, people make many mistakes when dealing with risk and delay, what results in many social problems, like debts, gambling, obesity, and many more. The presented literature suggests that instructing people to reflect on a problem from the perspective of an expert can significantly improve the performance. We expect our participants to produce less biased decisions in the field of risk and delay management.

The performance in risky condition is expected to be more consistent with the behavior predicted by EV normative model. This would provide an evidence of less biased risk assessment.

The performance in intertemporal choices is expected to change direction toward bigger patience. The ability to focus on bigger but more delayed goals seems to be more adaptive in modern society than is impulsivity; thus, our manipulation should boost patience. This assumption does not follow from any normative model, as in the case of risk; instead, the general social norm supports patient behavior rather than short-term oriented behavior (Haidt and Joseph, 2004). Hence, we expect participants to follow the social norm of impulsivity avoidance and produce lower discounting strength in the experimental condition in which participants are instructed to take the perspective of an expert.

## STUDY 1

We investigated the effect of forced hypothetical thinking on choices under risk. Situations, such as considering how much one is willing to invest to get a higher but uncertain return, are everyday problems, but the form of presenting the problem is relatively artificial, and people are evolutionally not prepared to deal with such problems. In other words, people can intuitively deal with risk, but the way they are presented increases the chance of making a biased decision (Gigerenzer and Selten, 2002).

By improving these types of judgment, we could enhance people’s entrepreneurship abilities as well as prevent risky, maladaptive behaviors, such as gambling or smoking. The hypothetical thinking can possibly increase the use of other competing intuitions or rule-based processing. Both should change the risk taking decision in a more normative manner and help people accurately estimate the EV of potential gains.

### MATERIALS AND METHODS

The certain equivalent of a potential gain of $5000 with a probability of 90, 70 and 30% was computed for every participant. The experimental manipulation was an instruction, asking participants to consider the problem from one of two perspectives, experts‘ or own. Our manipulation should increase abstract hypothetical thinking by taking the perspective of an expert^1^. Through this manipulation, we expected to debias human reasoning and discover the mindware responsible for risk management. The following is an example of tasks used:

Imagine you have received a $5000 reward, which will be paid to you with a 70% chance. An investment fund is willing to rebuy your reward with a certain payment. Imagine what an honest and rational expert would advise you to do – accept or refuse the given offer.

### ADJUSTING PROCEDURE

The research scheme used in all experiments was based on the adjusting method, which is most popular in the discounting research (Yi et al., 2006; Benzion et al., 2011; Odum, 2011). This procedure enables one to determine balance points, i.e., payment methods where the person being tested was indifferent to two given alternatives, for example, between receiving a sum x immediately and a sum y after a period of time t (in research on risky choices, between receiving a sum x for certain, and a sum y with a determined probability). Thanks to the adjusting method, the overestimation of expected gains can be prevented, which is common if individuals are asked directly, e.g., how much they would expect for having to wait for their gain.

The research was conducted using a specially developed computer program, which enabled us to determine the balance points. The characteristic feature of the adjusting method is that one choice alternative adjusts its value depending on previous decisions made by the person being tested. For example, during the test on risky payments, two cards depicting sums were displayed on the screen, one was bigger but uncertain (the card on the right side of the screen), and the other was smaller and certain (the card on the left side of the screen). The sum on the card on the right was fixed, while the card on the left changed its value depending on the subsequent choices of the person being tested.

The participant’s task was to choose between the two alternatives. With every question, the person being tested was to choose one of the values (certain or uncertain). In the first step, the participant was given a choice of $2500 for sure (information on the card on the left side) or $5000 with a 70% chance of winning (information on the card on the right side). After choosing the risky option, in the following step, the certain sum increased by half of its previous value. Hence, this time, the person being tested could choose $3750 for sure or $5000 with a 70% chance of winning. When the participant chose the risky option once more, the certain value increased by half of the previous value and amounted to $4375 ($3750 + $625) in the subsequent step. However, if in the next step ($4375 for sure or $5000 with a 70% chance of winning), the person being tested chose the certain sum, its value decreased by half of the previous change (by $312). To sum up, the certain value was adjusted to reflect previous choices of the person being tested. After making the sixth choice, the program calculated the equivalent point for the risky alternative.

### PARTICIPANTS

Participants (*n* = 105) were citizens of the United States recruited by specialized company in exchange for payment. They were randomly assigned to one of four experimental conditions. From this group, 27 participants who answered illogically (e.g., they wanted to receive more for a 30% chance of winning compared to 70% chance) were removed from the database prior to any further analysis. Mean age, gender distribution, and number of individuals in each experimental condition are presented in **Table 1**.

**Table 1:** Information about participants in Study 1.

Experimental condition	*N*	Ratio of females	Mean age (SD)
Expert perspective	39	38.5	34.077 (9.94)
Control group	39	43.6	33.58 (8.58)

Overall	78	41.0	33.8 (9.24)

### RESULTS

**FIGURE 1:**
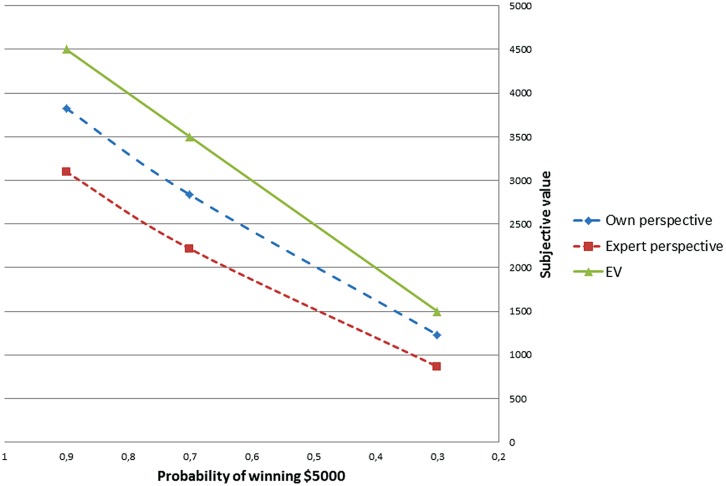
**Mean certain equivalents for own/expert perspective compared to expected value model**.

When a person is risk averse his subjective value of a lottery is lower. **Figure 1** presents a line that connects mean subjective values of each lottery computed for the own/expert perspective and compared to the EV model. We can see that individuals who took the perspective of an expert were more risk averse. Additionally, their result is further from the normative EV line compared to the control group individuals who solved the problem from their own perspective.

**FIGURE 2:**
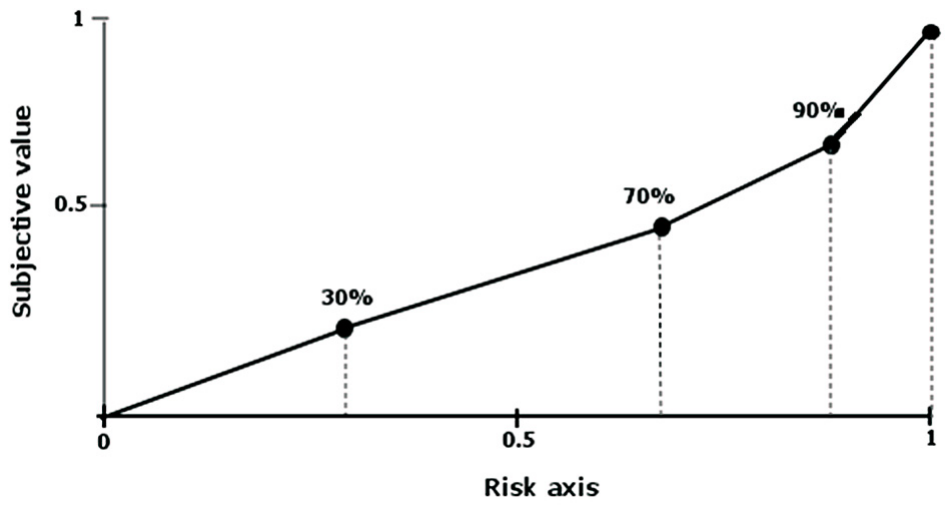
**The diagram shows four trapezes created as a result of connecting equivalent points, with the risk axis by line segments.** Both the risk and subjective value of the sum are standardized from the range from 0 to 1. Value 1 on the risk axis corresponds to a certain value, and on the subjective value axis the non-discounted sum. Own elaboration based on: Myerson et al. (2001).

To measure the attitude towards risk, the area under the curve was analyzed^2^ (Myerson et al., 2001). The surface of the area under the curve is the sum of all trapezes set by the next balance point values in relation to the ordinate and abscissa (**Figure 2**). By using this measure, one should first assign the values in the range (0, 1) to a scale of delay and the subjective value of the discounted values. In the case of the scale of delay, values are subsequently divided by the highest delays used. The scale of subjective values of the discounted sums is converted in a similar way. Next, we calculate the sum of the fields of the created trapezes using the formula (a2–a1) [(b1 + b2)/2], where a1 and a2 are consecutive delays while b1 and b2 represent consecutive subjective values of gains. The area under the empirical discounting curve is therefore the sum of all trapezes. The smaller the area under the curve the bigger is the risk aversion of an individual.

**FIGURE 3:**
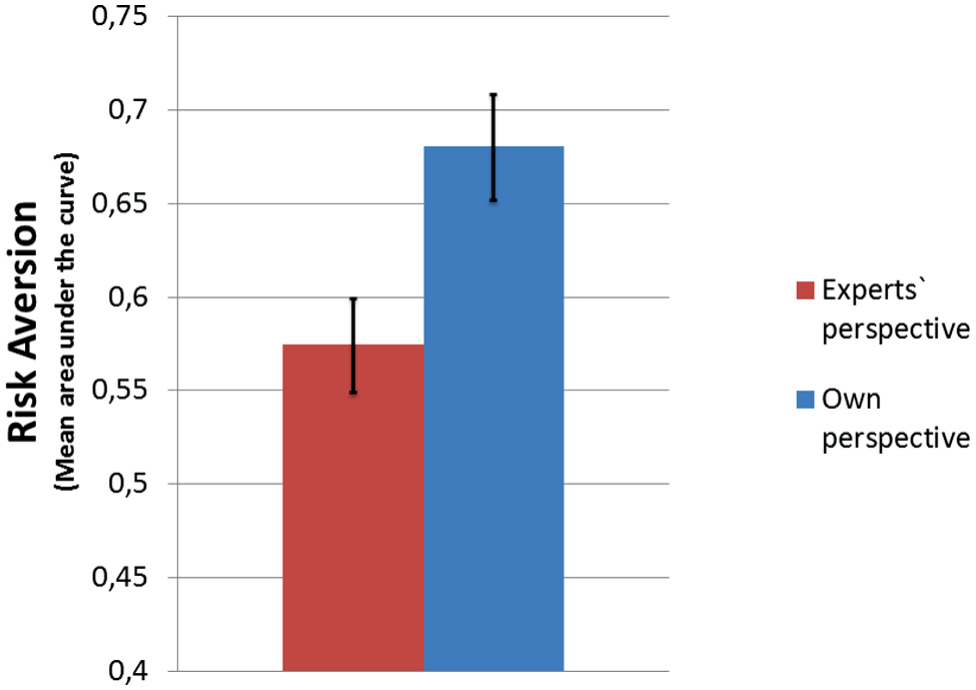
**Mean area under the curve for uncertain gains**.

Attitude toward risk was computed for each participant. Later, the parameters were compared across experimental groups. The general linear model showed the main effect of perspective [*F*(1,78) = 7.634; *p* < 0.01; η^2^ = 0.091]^3^, and the participants in the experimental group showed bigger risk aversion. The mean areas under the curve are shown in **Figure 3**. The bars represent the mean field under the curve, with higher value indicating more positive attitude toward risk (lower risk aversion).

### DISCUSSION

We found that taking the perspective of an expert significantly influenced the risky choices. Individuals in the experimental condition were more risk aversive. By comparing the choices in the study to the line representing the EV, we can conclude that the decisions made in the experimental condition were less normatively correct.

This result is contrary to our expectations because previously reported studies in other domains suggested that the perspective manipulation increases the normative correctness of decisions.

There are two possible explanations. First, the concept of EV is not known to participants or the social norm for risky decisions (risk avoidance) was made less salient thanks to the perspective shift. The first explanation seems less probable, as already children intuitively compare lotteries by multiplying gains and probabilities (Schlottmann, 2001). But if individuals would follow the social norm would expect to be blamed for unsuccessful risk taking and thus avoid doing so to greater extent.

Morsanyi and Handley (2012) showed that the use of Type 2 processing does not guarantee the correctness of thinking. Participants instructed to think from the perspective of a rational person focused even more on stereotypes instead of base rates when solving a lawyer’s task (Kahneman and Tversky, 1973).

In a study 1000 people were tested. Among the participants there were 5 engineers and 995 lawyers. Jack is a randomly chosen participant of this study. Jack is 36 years old. He is not married and is somewhat introverted. He likes to spend his free time reading science fiction and writing computer programs.

Most of individuals endorsed the conclusion that Jack is an engineer. They showed more interest in social stereotype than in the probability of occurrence of a specific event and answered against the odds. This case is especially interesting for understanding the naïve probabilities that humans calculate. Here, taking a rational perspective (Type 2 processing) exacerbated the neglect of base rates.

In other study, Pennycook et al. (2014) showed that the base rates are available at an intuitive level, so the increase of biased responses in referred Morsanyi and Handley study is a corrupted mindware case, where a social stereotype was judged as a more reliable source of information compared to the base rates. Additionally, in the field of risky decision, the instruction manipulation or enhancement of hypothetical thinking did not consistently improve the performance (Weinstein and Klein, 1995). The manipulation of instructions (hypothetical thinking) could possibly increase efficiency when one has the appropriate mindware: knows how to calculate the probability and how to use it in real-life social problems. If the mindware is missing, the performance can be even worse (Chiesi et al., 2011).

## STUDY 2

The aim of the second study was to investigate the effect of forced hypothetical thinking on intertemporal choices. Such situations are everyday problems, where one has to think about how much he/she is willing to invest now to get a higher but delayed return. By improving that type of judgment, we could persuade people to improve health or prevent such maladaptive behavior as overeating.

This assumption does not follow from any normative model, as in the case of risk, but the general social norm supports patience rather than short-term oriented actions (Haidt and Joseph, 2004). Thus, we expected participants to follow the social norm of impulsivity avoidance and produce lower discounting strength in the experimental condition of taking the perspective of an expert.

### MATERIALS AND METHODS

We repeated the procedure of Study 1. The only difference was that individuals had to evaluate three delayed gains. The delay was set for 1 month, 6 months, and 24 months. Once again, participants were randomly assigned to one of two experimental conditions, own perspective or the perspective of an expert. Additionally, in Study 2, we manipulated the ownership of the money (own money or someone else’s money), but this manipulation produced no main effect and did not interact with the perspective manipulation; thus, it will not be reported in following analysis.

### PARTICIPANTS

Participants (*n* = 130) were citizens of the United States recruited by external services in exchange for payment. They were randomly assigned to one of two experimental conditions. Details of the group are presented in **Table 2**. A small group of participants (*n* = 15) was excluded because of irrational responses (the same criterion as in Study 1 was used).

**Table 2:** Information about participants in Study 2.

Experimental condition	*N*	Ratio of females	Mean age (SD)
Experts perspective	62	35	34.4 (10.31)
Own perspective	53	57.9	37.5 (11.46)

Overall	115	46.15	35.91 (10.92)

### RESULTS

**FIGURE 4:**
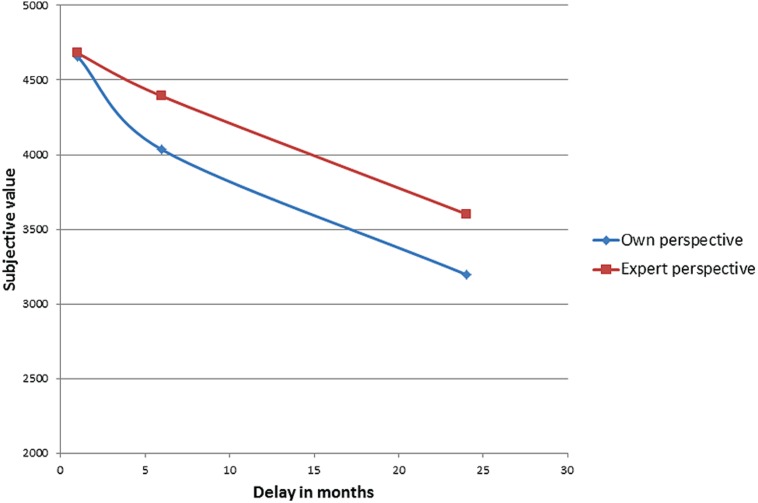
**Discounting curves for delayed gains**.

The discounting curves that connect balance points for delayed gain of $5000 are presented in **Figure 4**, from which we can see that when taking the perspective of an expert, people show less impulsivity compared to the control condition (own perspective).

**FIGURE 5:**
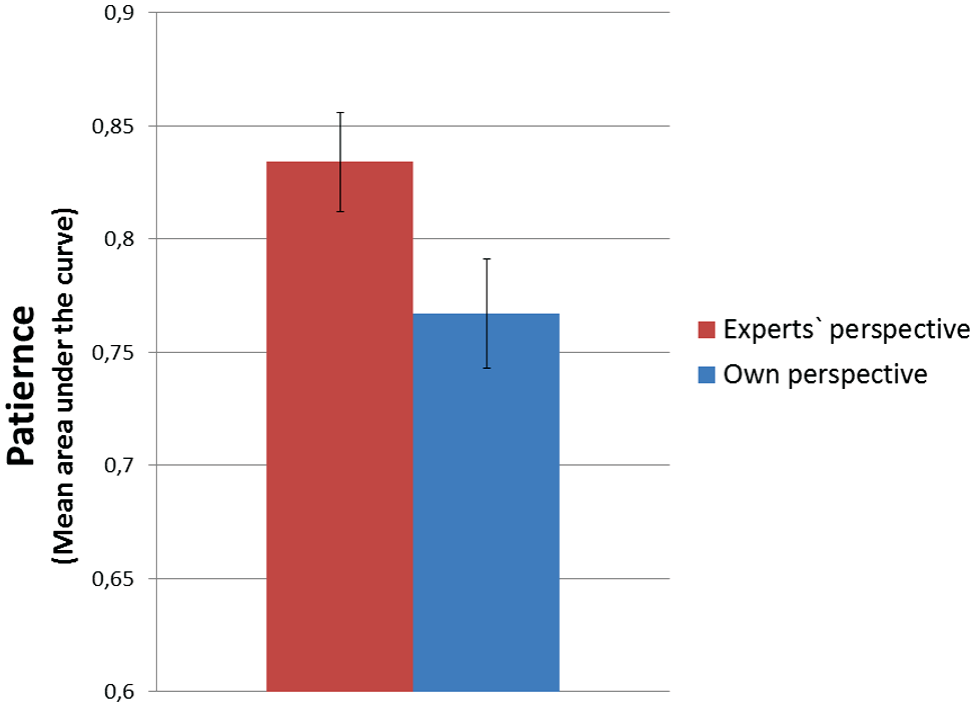
**Discounting strength for delayed gains**.

General linear model analysis was used to test the influence of possession and perspective on discounting strength. Once again, a main effect of perspective has been found [*F*(1,114) = 4.168; *p* < 0.05; η^2^ = 0.036]^4^. The mean discounting strength is presented in **Figure 5**.

### DISCUSSION

Taking the perspective of an expert improves thinking by helping individuals overcome impulsivity. Participants taking an expert’s perspective were less likely to lose some of their money by receiving it sooner and not having to wait compared to considering a problem from their own perspective. We can conclude that individuals’ mindware related to delay management is governed by a rule, where impulsivity is assessed as wrong, and the correct behavior is patience. This belief is then in conflict with the intuitive willingness to receive immediate rewards. This is consistent with common observations that people are sometimes consciously aware of the internal conflict between their intuitions (impulsivity, Type 1 processing) and beliefs about correct response (patience, Type 2 processing).

Despite the lack of normative model, we can conclude that the patience in this specific task presented here is adaptive; thus, it should be evaluated positively. This internal conflict should emerge when the mindware response is made salient by taking the perspective of an expert. De Neys (2014) stated that when the emotional arousal emerges after the conflict detection and an individual notices it, the heuristic response could be questioned. This can decrease the impulsivity, as it is a heuristic response.

## GENERAL DISCUSSION

The perspective shift changed human decisions. Participants showed bigger patience and risk aversion. The results in the area of risk are not consistent with our expectations. The decisions made cannot be seen as more correct or rational. The intertemporal choices have shown an improvement, as the discounting rate shown by individuals decreased. This can help individuals overcome temptations and help them make long-term financial plans (savings, investments).

Possible explanation of observed behavior is that individuals focused on social norms rather than on normative models. This would be consistent with findings of Chiesi et al. (2011) and Morsanyi and Handley (2012) who reported no consistent improvement or even decrease in correctness of decisions under the forced perspective shift. We discuss, that the experimental manipulation made the social norm salient and people who took experts’ perspective focused on the social norm to bigger extent, than in typical, everyday decision. Because patience and cautiousness are socially perceived as virtues we see a change of decisions to match those. This assumption is consistent with the reported findings on the improvement of thinking and reasoning presented in the introduction section. The social norm related to thinking promotes reflexive and logical thinking, that is, the Type 2 processing. The mindware responsible for dealing with risk is still a main topic for research on risk and delay management, but social norms related to this topic have to be investigated more and incorporated in the design of studies.

Our data did not fully support presented conclusion, as we have not tested the social norms of participants. We assume that the cultural norm should have some effect on most individuals. The proverb “A bird in the hand is worth two in the bush,” is a good example of socially accepted risk avoidance. The hypothesis of social norms made salient could be tested by comparing cultures with big differences in the delay management or in the attitude toward risk.

Despite possible difficulties, the idea of improving individuals’ decision-making in the area of risk and delay seems to be worth effort. Individuals perform sub-optimally even in advantageous conditions (with all required information provided and no time pressure) and require to be supported. Modern society created an artificial environment (e.g., by marketing, commercials) in which people are misinformed or put under time pressure; thus, human decision-making needs to be supported to greater extent, particularly by hypothetical thinking with the focus on a specific instruction.

## Conflict of Interest Statement

The authors declare that the research was conducted in the absence of any commercial or financial relationships that could be construed as a potential conflict of interest.

## Footnotes

1 We have also manipulated the ownership of the gain (deciding for myself or for somebody else’s gains), but it produced neither main effect nor interaction; thus, we do not report this manipulation in more details.

2 This measure has two significant advantages compared to an alternative analysis, which is based on comparing discounting parameters. First, it reduces the level of skewness compared to the analysis of the distribution of discounting parameters (k); second, it is neutral, i.e., it does not refer directly to any specific mathematical formula.

3 The analysis on full population of participants showed also a significant main effect of the perspective manipulation [*F*(1,104) = 5.187; *p* < 0.05; η^2^= 0.077]. By the elimination of irrational responses, we wanted to reduce a possible noise and report most reliable effect of the study.

4 As requested by reviewer, we tested gender as a factor in the GLM analysis and found no main effect or interaction (both *p* > 0.5).
